# Poly(C)-binding protein 1 mediates drug resistance in colorectal cancer

**DOI:** 10.18632/oncotarget.14516

**Published:** 2017-01-05

**Authors:** Jiani Guo, Changli Zhu, Kangqun Yang, Jin Li, Nan Du, Mingzhu Zong, Jianwei Zhou, Jingdong He

**Affiliations:** ^1^ Department of Oncology, Huai'an First People's Hospital, Nanjing Medical University, Huai'an, Jiangsu Province, China; ^2^ Department of Pharmacy, Huai'an First People's Hospital, Nanjing Medical University, Huai'an, Jiangsu Province, China; ^3^ Department of Molecular Cell Biology and Toxicology, Jiangsu Key Lab of Cancer Biomarkers, Prevention and Treatment, Cancer Center, Nanjing Medical University, Nanjing, China

**Keywords:** poly(C)-binding protein 1, colorectal cancer, chemoresistance, oxaliplatin

## Abstract

Oxaliplatin (L-OHP) is standard treatment for colorectal cancer. However, resistance to L-OHP often leads to treatment failure or cancer relapse. Understanding of the mechanism underlying L-OHP resistance is important to overcome the resistance and improve colorectal cancer treatment. This study aimed to identify new proteins that mediates L-OHP resistance in colorectal cancer and elucidate their mode of function. HT-29 cells were exposed to gradually increased concentration of L-OHP to select L-OHP resistant HT-29/L-OHP cell line. Proteomic analysis of HT-29 and HT-29/L-OHP cells were performed to identify differentially expressed proteins, including Poly(C)-binding protein 1 (PCBP1). PCBP1 expression level in 20 cases of L-OHP sensitive patients and 20 cases of L-OHP refractory patients was analyzed by immunohistochemistry. Chemoresistance and Akt activation in HT-29 and HT-29/L-OHP cells were analyzed by MTT assay and Western blot analysis. We identified 37 proteins showing differential expression in HT-29/L-OHP and HT-29 cells. In particular, PCBP1 protein level increased 15.6 fold in HT-29/L-OHP cells compared to HT-29 cells. Knockdown of PCBP1 sensitized HT-29/L-OHP and HT-29 cells to L-OHP, while overexpression of PCBP1 increased L-OHP resistance in HT-29 cells. In addition, PCBP1 expression was significantly higher in tumor samples from L-OHP refractory patients than in those from L-OHP responsive patients. Furthermore, we found that knockdown of PCBP1 inhibited the activation of Akt in HT-29/L-OHP and HT-29 cells. In conclusion, our findings suggest that PCBP1 is a molecular marker of L-OHP resistance in colorectal cancer and a promising target for colorectal cancer therapy.

## INTRODUCTION

Colorectal cancer is the third most common type of cancer in both men and women in the United States, and is the second leading cause of cancer-related mortality accounting for 9% of total cancer-related mortality in the United States [1]. Over the past two decades, overall colorectal cancer incidence and mortality rates have been declining, however, the prognosis of colorectal cancer remains poor. Identification of novel effective drugs or improvement of efficacy of anti-colorectal cancer drug would be very helpful for colorectal cancer treatment, especially for advanced colorectal cancer.

Oxaliplatin (L-OHP) is the third-generation platinum agent used as standard treatment for colorectal cancer, especially for advanced colorectal cancer [2]. Like other platinum-based anti-cancer compounds, L-OHP forms both inter- and intra-strand cross links in DNA as platinum (Pt)-DNA adducts, which prevent DNA replication and transcription, and cause cancer cell death. L-OHP is administered via infusion as inactive prodrug, and rapidly undergoes transformation in the blood to release cytotoxic dicholoro platinum complexes. L-OHP is often combined with other chemotherapeutic agents such as 5′FU to achieve synergistic effect, and can be used to treat some refractory colorectal cancers [2, 3]. However, resistance to L-OHP eventually develops, which leads to treatment failure or cancer relapse. Understanding of the mechanism underlying L-OHP resistance is very important to overcome the resistance and improve its efficacy in colorectal cancer treatment.

Several mechanisms have been suggested to explain L-OHP resistance. Besides efflux of the drug out of cell by plasma membrane pumps like MPR1, L-OHP could be detoxified by glutathione (GSH) related enzymes and Pt-DNA adducts lesions could be repaired by the nucleotide excision repair system (NER) [4]. ERCC1 and XPA are key mediators of NER and regulate L-OHP sensitivity [5–7]. DNA mismatch repair pathway (MMR) also participates in L-OHP resistance by removal of Pt-DNA adducts lesions. In this study, we systematically screened protein factors that might be involved in L-OHP resistance by the comparison of the proteome between L-OHP sensitive HT-29 wild type cells and L-OHP resistant HT-29 cells. Using the 2D gel electrophoresis and MALDI TOF/TOF tandem mass spectrometry, we identified 37 differentially expressed proteins. Among them, we found that poly(C)-binding protein 1 (PCBP1) was significantly induced in resistant cells, and our further study indicated that PCBP1 protected cells from L-OHP induced apoptosis. In addition, our analysis of clinical samples showed that PCBP1 was overexpressed in tumor samples from L-OHP resistant patients.

## RESULTS

### Establishment of L-OHP resistant HT-29 cells

**Figure 1:**
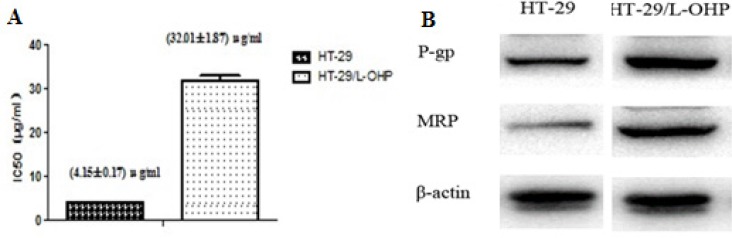
Establishment of L-OHP resistant HT-29 cell line (**A**) IC50 of HT-29 parental cells and HT-29/L-OHP cells. (**B**) Western blot analysis of MRP and P-gp expression in HT-29 parental cells and HT-29/L-OHP cells. β-actin was loading control.

To reveal molecular mechanism responsible for L-OHP resistance in colorectal cancer, wild type HT-29 cells sensitive to L-OHP were first treated with sub-lethal dose L-OHP to induce and select partial resistant populations. Then, L-OHP concentration was gradually increased and finally a population of L-OHP resistant HT-29/L-OHP cells were established. As shown in Figure 1A, the IC50 of parental wild type HT-29 was 4.15 ± 0.17 μg/mL, while the IC50 of L-OHP resistant HT-29/L-OHP cells increased to 32.01 ± 1.87 μg/mL, with drug resistance index of 7.71, indicating that HT-29/L-OHP cells were significantly resistant to L-OHP.

Multi-drug resistant protein (MRP) and P-glycoprotein (P-gp, ABCB1) are known mediators for drug resistance in cancer cells. As expected, we detected increased MRP and P-gp levels in HT-29/L-OHP cells compared to HT-29 parental cells (Figure 1B). Taken together, these results suggest that we successfully established L-OHP resistant HT-29 cell line.

### Screening of proteins that mediate L-OHP drug resistance

**Figure 2:**
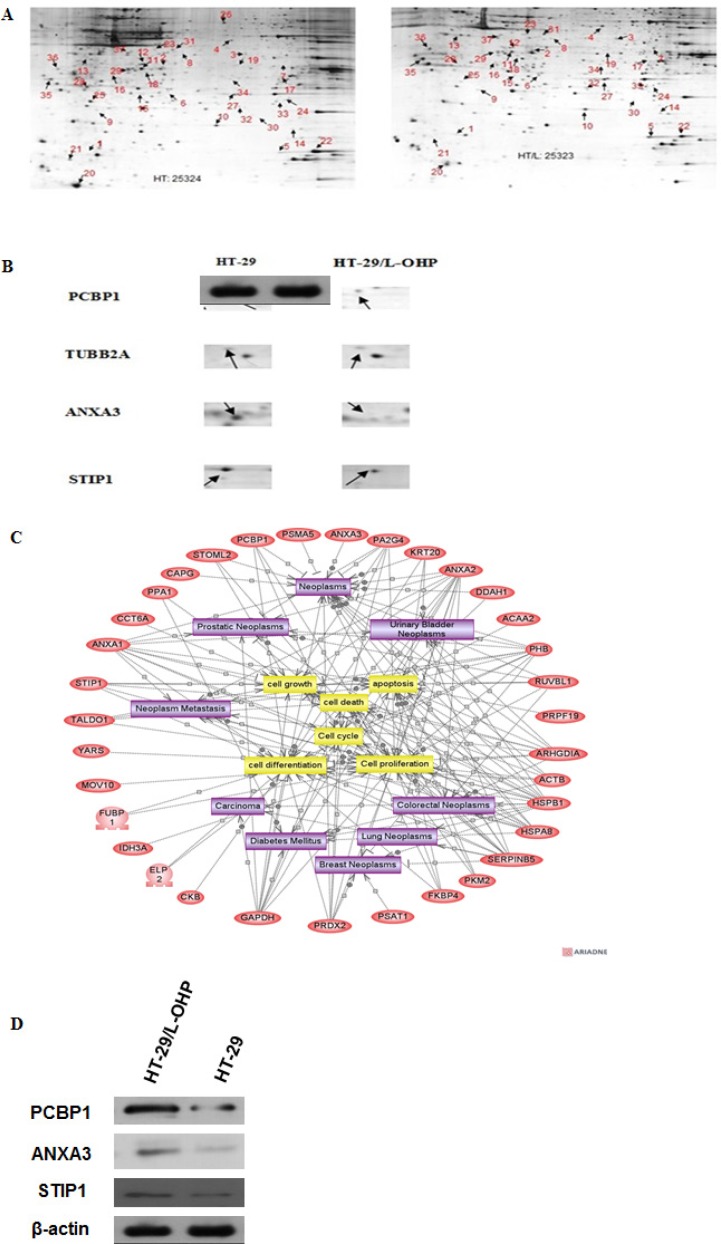
Proteomic analysis of proteins involved in L-OHP resistance in HT-29/L-OHP cells (**A**) 2D gel electrophoresis of HT-29 parental cells and HT-29/L-OHP cells. Arrows indicated protein spots with more than two fold expression difference between HT-29 parental cells and HT-29/L-OHP cells. (**B**) Enlarged view of protein spots of PCBP1, TUBB2A, ANXA3 and STIP1. (**C**) MALDI-TOF/TOF analysis of 37 identified proteins. **(D**) Western blot analysis of PCBP1, ANXA3, and STIP1 expression in HT-29/L-OHP cells. β-actin was loading control.

To screen proteins responsible for L-OHP drug resistance in HT-29/L-OHP cells other than MRP and P-gp, 2D gel electrophoresis (2DE) was performed to compare protein expression profile between HT-29 and HT-29/L-OHP cells. We identified 37 protein spots showing differential expression in HT-29/L-OHP and HT-29 cells (Figure 2A, 2B). The identities of these proteins were analyzed using MALDI TOF/TOF tandem mass spectrometry (MS). Protein function analysis showed that these proteins belonged to several functional groups: 1. Ca^2+^ binding proteins such as ANXA1, ANXA3; 2. chaperons such as HSPA1, HSPA8; 3. Metabolism such as GAPDH, PPA1, PKM2; 4. Cytoskeleton such as CAPZA1, CAPG, TUBB2A (Figure 2C). Identification of proteins involved in multiple cellular functions suggested that the alteration for drug resistance is complicated and involves multiple signaling pathways and cellular processes.

To confirm the results of 2DE and MS, we performed Western blot analysis to measure the protein expression in HT-29 and HT-29/L-OHP cells. As expected, PCBP1, ANXA3 and STIP1 protein levels increased in HT-29/L-OHP cells (Figure 2D). In particular, PCBP1 protein level increased 15.6 fold in L-OHP resistant cells than that in L-OHP sensitive parental cells, suggesting that PCBP1 could be a molecular marker of L-OHP resistance.

### PCBP1 is a molecular marker of L-OHP resistance in colorectal cancer

**Figure 3:**
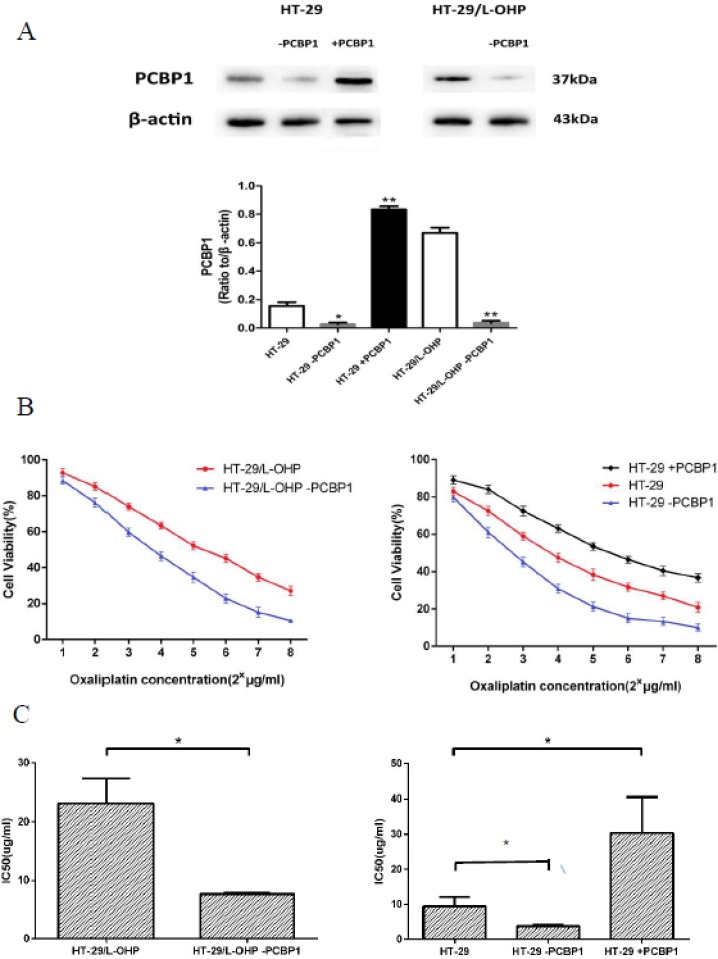
PCBP1 increased L-OHP resistance in HT-29 cells (**A**) Western blot analysis of PCBP1 levels in HT-29, HT-29+PCBP1, HT-29-PCBP1, HT-29/L-OHP and HT-29/L-OHP-PCBP1 cells. β-actin was loading control. *P < 0.05, **P < 0.01 vs. corresponding control. (**B**) Cell survival curve of HT-29/L-OHP cells with or without PCBP1 knockdown, and cell survival curve of HT-29 cells with or without PCBP1 knockdown and with exogenous PCBP1 expression. (**C**) IC50 of HT-29/L-OHP cells with or without PCBP1 knockdown, and IC50 of HT-29 cells with or without PCBP1 knockdown and with exogenous PCBP1 expression. *P < 0.05 vs. corresponding control.

PCBP1 is multifunctional RNA-binding protein that regulates mRNA processing, translation and stability [8]. PCBP1 also functions as a cytosolic iron chaperone that mediates the metalation of metalloproteins and enzymes, and help maintain cytosolic metal pools [9, 10]. To investigate the role of PCBP1 in L-OHP resistance, we modulated PCBP1 expression in HT-29/L-OHP and HT-29 cells by shRNA knockdown or exogenous expression of PCBP1 (Figure 3A). As shown in Figure 3B, knockdown of PCBP1 in HT-29/L-OHP cells significantly decreased IC50 in response to L-OHP. Knockdown of PCBP1 in parental HT-29 further sensitized the cells to L-OHP. In contrast, PCBP1 overexpression in HT-29 led to more than 3 fold increase of resistance to L-OHP (Figure 3C). These results demonstrated that PCBP1 increases L-OHP resistance in HT-29 cancer cells and may be a molecular marker of L-OHP resistance in colorectal cancer.

### PCBP1 is overexpressed in L-OHP resistant patient tumor samples

**Figure 4:**
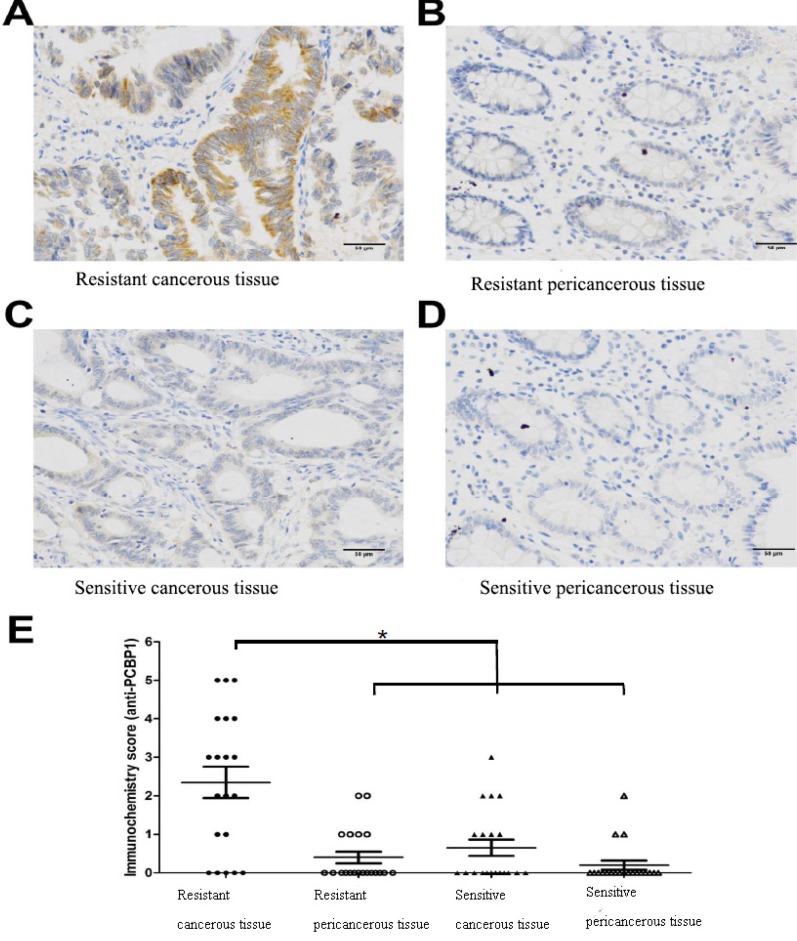
Higher PCBP1 expression in samples from L-OHP resistant patients (**A**) Representative strong staining of PCBP1 in tumor tissue from L-OHP resistant patient. (**B**) Representative weak staining of PCBP1 in peri-cancerous tissue from L-OHP resistant patient. (**C**) Representative weak staining of PCBP1 in tumor tissue from L-OHP sensitive patient. (**D**) Representative weak staining of PCBP1 in peri-cancerous tissue from L-OHP sensitive patient. Scale bar: 50 μm. (**E**) PCBP1 level was significantly higher in L-OHP resistant tumor tissue than that in L-OHP sensitive tumor tissue or peri-cancerous tissue (*P* < 0.05).

To provide *in vivo* evidence that increased PCBP1 expression is associated with L-OHP resistance, we analyzed 40 tumor samples from colorectal cancer patients among which 20 cases were L-OHP sensitive and 20 cases were L-OHP resistant. Immunochemistry analysis showed that PCBP1 protein level was high in L-OHP resistant patient tumor tissues (Figure 4A), but was very low in L-OHP resistant peri-cancerous tissues, L-OHP sensitive patient tumor tissues or L-OHP sensitive peri-cancerous tissues (Figure 4B–4D), and the difference in PCBP1 expression level between L-OHP resistant cancerous tissue and sensitive cancer tissue or peri-cancerous tissue was significant (*p* < 0.05). These clinical data supported that PCBP1 increases L-OHP resistance in colorectal cancer.

### PCBP1 enhances the activation of Akt

**Figure 5:**
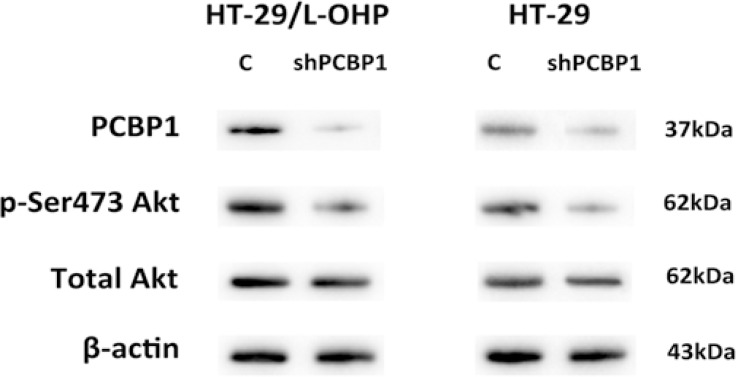
Knockdown of PCBP1 led to decreased Akt Ser473 phosphorylation in HT-29 and HT-29/L-OHP cells Western blot analysis of PCBP1, p-Ser473 Akt and total Akt levels in HT-29 and HT-29/L-OHP cells transfected with scramble siRNA (C) or PCBP1 siRNA (shPCBP1). Β-actin was loading control.

To understand how PCBP1 mediates L-OHP resistance in colorectal cancer, we focused on the effect of PCBP1 on cellular survival signaling pathways. Akt signaling pathway is one of important cell survival pathways that protect cells from cell death caused by many chemotherapy agents. Activation of Akt signaling promotes cell survival by phosphorylating and inactivating many components of the apoptotic machinery, such as Bad, caspase 9, and pro-apoptotic transcription factor FKHRL1 [11]. Therefore, we examined the phosphorylation of Akt Ser473 in both HT-29 parental and resistant cells after PCBP1 expression was silenced by shRNA. Knockdown of PCBP1 led to significantly decreased p-Akt level in both HT-29 parental and resistant cells, while the total Akt level showed no significant changes (Figure 5). These results indicated that PCBP1 enhances the activation of Akt to promote cell survival.

## DISCUSSION

Drug resistance is the major obstacle in cancer treatment. L-OHP is the first line drug for colorectal cancer treatment. However, resistance to L-OHP develops after long term usage, which leads to refractory tumor and/or cancer relapse. To understand the mechanism underlying L-OHP resistance in colorectal cancer, we established L-OHP resistant human colon cancer cell line by continuous exposure of HT-29 cells to L-OHP from sub-lethal concentration to gradually increased high concentration. The IC50 of L-OHP resistant HT-29/L-OHP cell line was increased more than 8 fold (from 4.15 ± 0.17 μg/mL to 32.01 ± 1.87 μg/mL). In addition, increased expression of multi-drug resistant genes MRP1 and P-gp was detected in HT-29/L-OHP cell line, indicating that we successfully established L-OHP resistant colorectal cancer cell line as a nice experimental model for further investigation of L-OHP resistance in colorectal cancer.

Next, we systematically investigated the proteins involved in L-OHP resistance in HT-29/L-OHP cells by using 2D gel electrophoresis followed by MALDI TOF/TOF tandem mass spectrometry. We identified 37 proteins that were differently expressed in L-OHP resistant versus sensitive cells. Protein function analysis showed that these proteins had many different cellular functions, including Ca^2+^ binding, molecular chaperons, metabolism and cytoskeleton, which suggest that the resistant cells undergo profound changes of expression profiles to gain L-OHP resistance. It was reported that increased DNA damage repair capability is an approach to enhance L-OHP resistance by removing L-OHP caused DNA damages through nucleotide excision repair and/or mismatch repair pathways [6, 7]. Our results demonstrate that there are many other cellular pathways involved in L-OHP resistance, suggesting that L-OHP resistant cancer cells are not merely dependent on one or two pathways but undergo complicated evolution and impact complex pathways to achieve resistance. Therefore, understanding of all these pathways involved in resistance becomes the key to overcome L-OHP resistance, since targeting one or two pathways will not completely or effectively inhibit the resistance.

Among 37 identified proteins, we confirmed increased expression of PCBP1, ANXA3 and STIP1 in resistant cells by Western blot analysis. Especially, PCBP1 level was increased 15.6 fold in L-OHP resistant cells compared to L-OHP sensitive parental cells. PCBP1 is a multifunctional adaptor protein initially identified as a RNA-binding protein [8, 12]. PCBP1 contains three K-homologous (KH) domains and has high affinity to poly(C) [12]. PCBP1 and its homologs can bind to mRNA 3′UTR pyrimidine-rich motif to stabilize mRNA and regulate mRNA translation [13–17]. In addition, PCBP1 functions as cytosolic iron chaperone that facilitates iron delivery to ferritin, the major cytosolic iron storage protein, to metalloproteins, prolyl and asparagyl hydroxylases, which regulate the stability of the α-subunit of hypoxia-inducible factor (HIF), and to deoxyhypusine hydroxylase (DOHH), which modifies hypusine in eukaryotic initiation factor (eIF) 5A [9, 10, 18]. Our results showed that knockdown of PCBP1 expression in both wild type and L-OHP resistant HT-29 cells sensitized cells to L-OHP, while overexpression of PCBP1 in HT-29 wild type cells increased L-OHP resistance. Additionally, by comparison of PCBP1 expression in tumor samples from L-OHP responsive and refractory patients, we found that PCBP1 expression was significantly higher in tumor samples from L-OHP resistant patients. Taken together, these results demonstrate that PCBP1 plays an important role in L-OHP resistance and can be served as a molecular marker for L-OHP resistance in colorectal cancer patients.

To further elucidate how PCBP1 mediates L-OHP resistance, we investigated cell survival signaling pathways and found that the activation of Akt signaling was linked to increased expression of PCBP1. Interestingly, Akt2 was reported to be able to phosphate PCBP1 and regulate its activity [17, 19, 20], thus indicating a strong connection between PCBP1 and Akt signaling pathway. PCBP1 might protect cells from L-OHP by enhancing cell survival pathway such as Akt signaling to prevent L-OHP induced cell death. Future studies are needed to characterize the interaction between PCBP1 and Akt, and their contribution to drug resistance in cancer therapy.

## MATERIALS AND METHODS

### Cell culture

Human colorectal cancer cell line HT-29 was purchased from American Type Culture Collection (ATCC, Manassas, VA, USA) and cultured in Dulbecco's modified Eagle's medium (DMEM, Gibco, Carlsbad, CA, USA) medium supplemented with 10% fetal bovine serum (FBS, Gibco) at 37°C with 5% CO2. To establish L-OHP resistant cell line, HT-29 cells in the exponential phase of growth were exposed to 50 ng/ml L-OHP for 1 month. L-OHP resistant HT-29/L-OHP cell line was established three months after the drug treatment was initiated and then maintained in L-OHP free medium and subcultured for at least 3 times.

### 2D gel electrophoresis

HT-29 and HT-29/L-OHP cells were harvested and washed with 1 × PBS once, then lysed with lysis buffer containing 1 × protease inhibitor cocktail. The lysate was centrifuged at 14,000 rpm for 10 min and the supernatants were collected. Total protein lysate was first loaded into pH gradient gel to be separated by isoelectric focusing. Next, the proteins in pH gradient gel were separated by SDS-PAGE. Then the gels were stained to visualize protein spots. The protein spots with difference between HT-29 and HT-29/L-OHP cells were identified and excised from the gels for mass spectrometry.

### MALDI TOF/TOF tandem mass spectrometry

The protein spots were dried by vacuum and digested by incubation with trypsin solution overnight. Tryptic peptides were eluted and dried by vacuum. The tryptic peptides were analyzed by AutoFlex TOF-TOF LIFT Mass Spectrometer and the corresponding proteins were identified by using the Mascot search engine against NCBInr protein database.

### Western blot analysis

Cells were harvested and lysed with lysis buffer containing protease inhibitor cocktail and phosphatase inhibitor cocktail. 50 μg total protein lysate were loaded on SDS-PAGE gel and transferred to PVDF membranes. The membranes were blocked with 5% non-fat milk for 1 h, incubated with primary antibody for PCBP1, p-Akt Ser473, Akt, ANXA3, STIP1, GAPDH, P-gp, MRP1, β-actin antibodies (Santa Cruz Biotech, Santa Cruz, CA, USA) overnight at 4°C, then washed with 1 × TBS/T 3 times, 5 min each time. The membranes were then incubated with secondary antibody diluted in 1 × TBS/T with 5% non-fat milk for 1 h at room temperature. After washing with 1 × TBS/T 3 times, ECL reagent was added to visualize the signal and the signal was analzyed by gel imager.

### MTT assay

HT-29 or HT-29/L-OHP cells were seeded into 96-well plates and cultured in a humidified chamber at 37°C overnight. Next the cells were treated with L-OHP at 1 to 8 μg/mL. Then viable cells were evaluated with MTT assay kit (Sigma, USA) according to the manufacturer's instructions. 20 μL MTT (5 mg/mL) solution was added to each well and the plates were incubated at 37°C for 4 h, then 150 μL DMSO was added to each well and the plates were incubated at room temperature for 10 min. The absorption value was read at 490 nm using a microplate reader (ELX800, Bio-Tek, USA).

### Immunohistochemistry

The response of patients to L-OHP was evaluated according to Response Evaluation Criteria in Solid Tumors (RECIST) criteria. Tumor samples were excised from 20 cases of L-OHP sensitive patients and 20 cases of L-OHP refractory patients, fixed in formalin, embedded in paraffin and cut into 4 μm serial sections. Next, endogenous peroxidase was quenched and the sections were incubated with 2% goat serum at room temperature for 1 h, then incubated with PCBP1 antibody (Santa Cruz Biotechnology, USA) overnight at 4°C. Afterwards, the sections were incubated with horseradish peroxidase-conjugated secondary antibody. For negative control, IgG serum was used instead of PCBP1 antibody.

Two experienced pathologists blindly evaluated immunohistochemistry score. The percentage of cells with staining was scored as:”0” (0%), “1” (1%–25%), “2” (26%–50%), “3” (51%–100%). The staining intensity was scored as:”0” (negative), “1” (weak), “2” (moderate), “3” (strong). Immunohistochemistry score was obtained by adding the two scores.

### Statistical analysis

Data were represented as the mean ± SD and analyzed using SPSS version 12 statistical analysis package (SPSS Inc., Chicago, IL, USA). *P* < 0.05 was considered statistically significant.
